# Neuromedin U secreted by colorectal cancer cells promotes a tumour-supporting microenvironment

**DOI:** 10.1186/s12964-022-01003-1

**Published:** 2022-12-08

**Authors:** Patrycja Przygodzka, Kamila Soboska, Ewelina Sochacka, Marcin Pacholczyk, Marcin Braun, Hassan Kassassir, Izabela Papiewska-Pająk, Michal Kielbik, Joanna Boncela

**Affiliations:** 1 Institute of Medical Biology, Polish Academy of Sciences, Lodowa 106, 93-232 Lodz, Poland; 2 Faculty of Biology and Environmental Protection, University of Lodz, Pomorska 141/143, 90-236 Lodz, Poland; 3 Department of Systems Biology and Engineering, Silesian University of Technology, Akademicka 16, 44-100 Gliwice, Poland; 4 Department of Pathology, Medical University of Lodz, Pomorska 251, 92-213 Lodz, Poland

**Keywords:** Colorectal cancer, Neuromedin U, NMU receptors, Macrophages, Endothelial cells

## Abstract

**Background:**

Neuromedin U (NMU) was identified as one of the hub genes closely related to colorectal cancer (CRC) progression and was recently shown to be a motility inducer in CRC cells. Its autocrine signalling through specific receptors increases cancer cell migration and invasiveness. Because of insufficient knowledge concerning NMU accessibility and action in the tumour microenvironment, its role in CRC remains poorly understood and its potential as a therapeutic target is still difficult to define.

**Methods:**

NMU expression in CRC tissue was detected by IHC. Data from The Cancer Genome Atlas were used to analyse gene expression in CRC. mRNA and protein expression was detected by real-time PCR, immunoblotting or immunofluorescence staining and analysed using confocal microscopy or flow cytometry. Proteome Profiler was used to detect changes in the profiles of cytokines released by cells constituting tumour microenvironment after NMU treatment. NMU receptor activity was monitored by detecting ERK1/2 activation. Transwell cell migration, wound healing assay and microtube formation assay were used to evaluate the effects of NMU on the migration of cancer cells, human macrophages and endothelial cells.

**Results:**

Our current study showed increased NMU levels in human CRC when compared to normal adjacent tissue. We detected a correlation between high *NMUR1* expression and shorter overall survival of patients with CRC. We identified NMUR1 expression on macrophages, endothelial cells, platelets, and NMUR1 presence in platelet microparticles. We confirmed ERK1/2 activation by treatment of macrophages and endothelial cells with NMU, which induced pro-metastatic phenotypes of analysed cells and changed their secretome. Finally, we showed that NMU-stimulated macrophages increased the migratory potential of CRC cells.

**Conclusions:**

We propose that NMU is involved in the modulation and promotion of the pro-metastatic tumour microenvironment in CRC through the activation of cancer cells and other tumour niche cells, macrophages and endothelial cells.

**Video abstract**

**Supplementary Information:**

The online version contains supplementary material available at 10.1186/s12964-022-01003-1.

## Background

Colorectal cancer (CRC) is one of the leading causes of cancer-related deaths worldwide, with 1.9 million incidence cases and 0.9 million deaths reported worldwide in 2020 [1]. In recent years, the introduction of screening programs for CRC has resulted in an increased number of CRC cases [2]. However, effective treatment is challenging, even when diagnosed early, because of intertumoral [3] and intratumoral [4] heterogeneity. An understanding of the molecular mechanisms that increase cancer cell invasiveness and the identification of biomarkers for a poor prognosis are still critical to tumour staging and choosing therapy for patients with CRC.

Neuromedin U (NMU), a small secreted peptide, has a myriad of different functions [5] and recently became a point of interest in cancer studies [6]. It is already considered a tumour growth and/or progression marker in endometrial [7], renal [8], and breast cancers [9] but its role in CRC as well as its potential as a therapeutic target are still difficult to define. NMU acts through receptors that belong to the superfamily of G protein-coupled receptors (GPCRs), including primary receptors, i.e., NMUR1 and NMUR2, and alternative receptors, i.e., NTSR1/GHSR1b heterodimers [6]. These transmembrane receptors are distributed in various tissues and cells, including the central nervous system, gastrointestinal tract [10], cardiovascular system [11] and immune cells [12]. Both NMUR1 and NMUR2 share high homology, and they have been reported to activate the same signalling pathways involving inositol phosphates and calcium ions as secondary messengers [6].

Our previous studies identified the upregulation of *NMU* in invasive CRC cells [13]. An analysis of patient datasets (collected in The Cancer Genome Atlas, TCGA; http://tcga-data.nci.nih.gov/tcga/) enabled us to report significantly higher *NMU* and *NMUR2* expression and lower *NMUR1* expression in primary CRC tissues than in normal epithelium [14]. We also showed that the autocrine action of NMU through NMUR2 activation on CRC cells leads to a prometastatic phenotypic switch [14].

Here, we continue to research the roles of NMU and NMURs in CRC progression. According to our analysis of TCGA datasets, *NMUR1* expression appeared to correlate with worse patient outcomes. In addition to CRC cells [14], many other cells present in the tumour microenvironment (TME) express NMUR1 [12]; therefore, in addition to its autocrine activity, a paracrine effect of NMU released by CRC cells is expected. Here, we analysed cancer cells, as well as macrophages, endothelial cells and platelets, which are known for their capability to modulate cancer invasiveness, as potential NMU targets present in the TME.

## Materials and methods

### NMU peptide

NMU-9 (Phoenix Pharmaceuticals, Burlingame, CA, USA) has a comparable affinity to NMU-25 for human NMU receptors, with an exact half-life in human plasma t1/2 = 4.60 ± 0.4 min [14, 15]. NMU-9 contains nine C-terminal amino acids of human NMU-25 along with a highly conserved, amidated pentapeptide at the C-terminus determining peptide bioactivity.

### Immunohistochemical staining of tissue arrays and data analysis

Immunohistochemical staining for NMU was evaluated using the preoptimized protocol described below. A rabbit anti-NMU antibody (Sigma–Aldrich, Saint Louis, MO, USA) was used, and immunohistochemical staining was performed on commercially available colorectal cancer tissue microarrays (TissueArray.Com LLC, Derwood, MD, US previously US Biomax). Antigens were unmasked, stained and visualized using an EnVision FLEX, High pH (Link) (Dako-Agilent, San Jose, CA, USA) detection system according to the manufacturer’s instructions. Nuclei were counterstained with haematoxylin. A positive control, pheochromocytoma, was included on each tissue microarray. Negative control slides were stained with rabbit IgG antibodies (Sigma–Aldrich). Next, the slides were digitalized using an UltraFast Scanner (Philips IntelliSite Pathology Solution, USA). The NMU protein level was evaluated by an expert in pathology (M.B.). Images of representative samples were captured using DigiPath™ Professional Production Software (Xerox, Norwalk, CT, USA). The NMU protein level in each slide was evaluated semiquantitatively by applying a modified 0–300 H-score scale [16]. NMU intensity was evaluated on a 0–3 scale, the percentages of epithelial/cancerous cells positive for NMU (among all epithelial/cancerous cells of the respective slide) were quantified, and intensity scores were multiplied by percentage levels for each slide. Cases with final scores of 0 to 10 were annotated as negative for NMU expression, whereas cases with scores > 10 were annotated as positive (10–75 as weakly positive; 75–125 as moderately positive and > 125 as strongly positive).

### TCGA patient dataset

We downloaded gene expression data (RNA Seq v2) as RSEM normalized logarithms of raw counts from TCGA colorectal adenocarcinoma dataset (TCGA_coad) using FirebrowseR package in R. We analysed 621 tumour samples. The data were further divided into different groups according to selected clinical attributes: tumour stage, tumour site, metastasis indicator, etc. Overall survival (OS) was measured from surgery until death and was censored for patients who were alive at the last follow-up.

### Cell culture

The human colon carcinoma cell lines HT29 and HCT116, the human monocytic cell line THP-1 and the microvascular endothelial cell line HMEC-1 were obtained from the American Type Culture Collection (ATCC, Manassas, VA, USA). The HT29-pcNMU clone overexpressing NMU and the corresponding control clone HT29-pcDNA were generated and characterized previously [14]. HCT116 cells were cultured in McCoy’s 5A medium (Thermo Fisher Scientific, Waltham, MA, USA), THP-1 cells were cultured in RPMI 1640 medium (with the ATCC modification, Thermo Fisher Scientific) supplemented with 0.05 mM β-mercaptoethanol, HMEC-1 cells were cultured in MCDB131 medium supplemented with 10 ng/ml epidermal growth factor (EGF; Thermo Fisher Scientific), 1 µg/ml hydrocortisone (Sigma–Aldrich) and 5 mM L-glutamine (Sigma–Aldrich), and HT29 clones were cultured in RPMI 1640 medium (with ATCC modification) supplemented with 400 μg/ml hygromycin B (Thermo Fisher Scientific). All media were supplemented with 10% fetal bovine serum (FBS; Sigma–Aldrich) (for HCT116 cells, the FBS was heat-inactivated), penicillin/streptomycin (pen/strep) (Thermo Fisher Scientific) and primocin (InvivoGen, San Diego, CA, USA). Cells were cultured at 37 ˚C in an incubator containing 5% CO_2_ and 95% humidified air. Cells were routinely tested for Mycoplasma (PlasmoTest; InvivoGen). Routine authentication of the cell lines was performed (Eurofins, Luxemburg) (Additional file 1).

### Human monocyte isolation

Peripheral blood mononuclear cells (PBMCs) were isolated by centrifugation (500 × g, 30 min, RT) in a Histopaque-1077 density gradient (Sigma–Aldrich) of buffy coats obtained from healthy donors from Regional Centre of Blood Donation and Blood Treatment in Lodz in accordance with applicable law. Peripheral blood monocytes (PBMs) were purified from the PBMC fraction by magnetic separation on MACS MS columns (Miltenyi Biotec, Germany) using MACS CD14 MicroBeads according to the manufacturer’s protocols. PBMs were maintained in RPMI 1640 medium (with ATCC modification) supplemented with 10% human AB serum (Sigma–Aldrich) and pen/strep for use in subsequent procedures.

### Monocyte differentiation and macrophage polarization

MDMs (monocytes-derived macrophages) were obtained by incubating PBMs with 10 ng/ml GM-CSF (Thermo Fischer Scientific) for 7 days with medium exchange every 48 h. TDMs (THP-1-derived macrophages) were obtained by incubating THP-1 cells with 20 ng/ml phorbol 12-myristate 13-acetate (PMA; InvivoGen) for 2 days, followed by an incubation in full medium for 24 h. MDM M0 and TDM M0 cells were polarized into the M1 phenotype by an incubation with 20 ng/ml IFN-γ (R&D Systems, Minneapolis, MN, USA) and 10 pg/ml LPS (Sigma–Aldrich) for 5 days and 1 day, respectively. MDM M0 cells were polarized for 5 days and TDM M0 cells were polarized for 4 days into the M2a phenotype with 20 ng/ml IL-4 (R&D Systems) or into the M2c phenotype with IL-10 (R&D Systems). All phenotypes were verified by detecting the presence of cell surface markers using flow cytometry (Additional file 2: Fig. S1).

### Conditioned media (CM) preparation

PBMCs (2 × 10^6^) cultured in 6-well plates were differentiated into macrophages using the procedures described above. HMEC-1 cells were grown on 6-well plates in growth medium to 70–80% confluence. Then, the cells were cultured for 24 h without GM-CSF (PBMCs) or hydrocortisone and EGF (HMEC-1). Next, the cells were washed with PBS, and 1.3 ml of fresh medium (RPMI 1640 supplemented with 10% human serum AB and MCDB131 medium containing 5% FBS for cytokine detection or RPMI 1640 supplemented with 5% FBS for the CRC migration assay) was added and conditioned for 24 h with or without 500 nM NMU-9. Finally, conditioned media were collected, centrifuged (1000 × g, 20 min, 4 °C for cytokine detection and RT for the CRC migration assay) and directly used in subsequent experiments. HT29-pcNMU and control HT29-pcDNA CM were prepared as previously described [14].

### mRNA isolation and real-time PCR analysis

Total RNA was isolated with the ReliaPrep™ RNA Cell Miniprep System (Promega, Madison, WI, USA). Quality control of the isolated RNA was performed using a 2100 Bioanalyzer (Agilent Technologies, Palo Alto, CA, USA) according to the manufacturer's instructions. Then, 0,5 or 1 µg of the isolated total RNA (RIN ≥ 8) was reverse transcribed using a High-Capacity cDNA Reverse Transcription Kit (Applied Biosystems, Waltham, MA, USA) according to the manufacturer's instructions. Real-time PCR for human *NMU, NMUR1, NMUR2, NSTR1, GHSR1b, GAPDH* and *Actβ* was performed using FastStart Essential DNA Green Master Mix (Roche, Basel, Switzerland) and previously designed primers [14]. Amplification was performed on a Roche LightCycler 96. *GADPH* and/or *β-actin* mRNA transcripts were used as internal controls. The amount of target in the various samples was calculated using the 2^−ΔCt^ relative quantification method with DataAssist v.3.01.

### Western blot

Cells were lysed in RIPA buffer (100 mM Tris–HCl, pH 7.5, 300 mM NaCl, and 0.2% SDS) containing 1% IGEPAL CA-630 and the Halt™ Protease Inhibitor Cocktail (Thermo Fisher Scientific) with subsequent centrifugation (18,000 × g, 4 °C, 20 min). The total protein concentration in the supernatants was quantified using the BCA method (Pierce BCA Protein Assay; Thermo Fisher Scientific). Samples with equal total protein content were loaded and separated on SDS–PAGE gels (Mini-PROTEAN TGX Stain-Free Gels; Bio-Rad Laboratories, Hercules, CA, USA) and then transferred onto nitrocellulose membranes (Bio-Rad Laboratories). We used the following primary antibodies: rabbit anti-NMU, rabbit anti-NMUR1 (Genetex, Irvine, CA, USA), mouse anti-IGFBP-7 and mouse anti-vimentin (Santa Cruz Biotechnology, Dallas, TX, USA). For the analysis of ERK1/2 levels, mouse anti-ERK1/2 (Santa Cruz Biotechnology) and rabbit anti-pERK1/2 antibodies (Thermo Fisher Scientific) were used. GAPDH or α-tubulin was used as a loading control and detected using rabbit anti-GAPDH or mouse anti-α-tubulin antibodies, respectively (Abcam, Cambridge, GB). HRP-conjugated goat, anti-mouse (Santa Cruz Biotechnology) or anti-rabbit secondary antibodies (Thermo Fisher Scientific) were used. The signal was detected by measuring the chemiluminescence (Thermo Fisher Scientific) with Kodak BioMax Light Film from Eastman Kodak (NY, USA).

### Immunofluorescence staining and confocal microscopy

PBMs, THP-1 cells (1.0 × 10^5^), or HMEC-1 cells (1.5 × 10^5^) were seeded on 0.1 mg/ml poly-D-lysine-coated glass 8-well chamber slides (Thermo Fisher Scientific) and incubated in cell-specific culture medium for 24 h to adhere. Macrophages were differentiated from 0.5 × 10^5^ of PBMs or THP-1 cells. For immunofluorescence staining, cells were fixed by a 10-min incubation with 4% paraformaldehyde and blocked with 5% BSA in PBS for 30 min. NMUR1 was detected by a 1 h incubation with rabbit anti-NMUR1 antibodies (Genetex) followed by a 30 min incubation in the dark with Alexa-Fluor-488-conjugated donkey anti-rabbit secondary antibodies (Thermo Fischer Scientific) (both diluted with PBS containing 3% BSA). Cell membranes and nuclei were visualized by a 10-min incubation in the dark with 2 μM Alexa-Fluor 594-conjugated wheat germ agglutinin (WGA) and 5 μg/ml Hoechst 33342 (in 3% BSA in PBS) respectively. Stained cells were visualized using a confocal microscope (Nikon D-Eclipse C1) and analysed with EZ-C1 version 3.6 software (Nikon, Japan).

### ERK1/2 activation

A total of 1.5 × 10^6^ THP-1 cells or PBMs were differentiated into macrophages, and HMEC-1 cells were grown to 70–80% confluence in 6-well plates. Twenty-four hours prior to the experiment, the cell-specific culture media were changed to media without supplements except for FBS and pen/strep, and then the cells were starved (without FBS) for 6 h prior to the experiment. Subsequently, cells were treated with 250 nM NMU-9 or HT29 CM for 3, 7, 15 or 30 min or 3 ng/ml PMA for 15 min as a positive control. Cell lysates were prepared as described above, and 10 µg of total protein were loaded and separated on 10% SDS–PAGE gels and analysed using Western blotting.

### Flow cytometry

The levels of surface markers of differentiation (CD11b or CD14) and polarization (HLA-DR and CD80 for M1 or CD206 and CD163 for M2a and c phenotypes) were assessed in THP-1 cells/PBMs and TDMs/MDMs of different phenotypes. Selected markers of M1 (HLA-DR) and M2 (CD206) phenotypes were analysed in MDMs treated with 500 nM NMU-9 (for 24 h) using immunofluorescence staining and flow cytometry. Macrophages were detached on ice with cold 0.5% BSA in PBS with 2 mM EDTA, resuspended in PBS/0.5% BSA/2 mM EDTA and stained with fluorophore-conjugated antibodies (all from BD Biosciences, Franklin Lakes, NJ, USA, described in Additional file 2: Table S1) for 30 min at RT in the dark. A washing step with PBS containing 2 mM EDTA was performed. The FACS analysis was performed using a FACS LSR II BD flow cytometer (Becton Dickinson) equipped with BD FACS Diva Software. The results were analysed using FlowJo™ 10.7.1 software (BD).

### Cytokine secretion

The levels of cytokines secreted by cells upon NMU treatment were quantified in conditioned media using a Proteome Profiler Human Cytokine Array Kit (R&D Biosystems) according to the manufacturer’s instructions. The cytokine spot intensity was analysed using ImageJ 1.53e [17] software and normalized to the mean reference intensity and to the protein level in CM.

### Transwell cell migration assay

Macrophage migration assays were performed using Transwells with 5.0 μm pore polycarbonate membrane inserts (Costar/Corning). A total of 1.5 × 10^6^ THP-1 or PBM cells was differentiated using a standard procedure in 6-well plates, incubated for 24 h prior to the experiment without PMA or GM-CSF, respectively, and preincubated with 500 nM NMU-9 or HT29 CM for 3.5 h. Macrophages were detached on ice with cold PBS containing 0.5% BSA and 2 mM EDTA, and 1.4 × 10^5^ cells were resuspended in 200 µl of cell culture medium supplemented with 0.1% FBS and seeded in the insert placed in the well with 600 µl of the same medium. Cells were incubated for 1 h and 45 min (TDMs) or 3.5 h (MDMs) under standard cell culture conditions. Inserts were fixed with cold methanol and acetic acid (3:1) for 10 min at − 20 °C, and cells remaining inside the chamber were gently removed with cotton swabs. Cells on the lower side of the insert were stained with 0.5% crystal violet for 30 min, and the filters were washed with PBS. Images of 5 randomly chosen fields were captured under a Nikon Eclipse TE 2000-U microscope, and migratory cells were counted.

### Wound healing cell migration assay

HMEC-1 or HCT116 cells were grown in complete culture medium in 0.1 mg/ml poly-D-lysine-coated 24-well plates until reaching confluence. For HMEC-1 cells, twenty-four hours prior to the experiment, the medium was changed to MCDB-131 culture medium without EGF and hydrocortisone, and then the cells were preincubated for 3.5 h with 500 nM NMU-9 or 50 ng/ml VEGF-A (R&D Systems) in MCDB131 medium (supplemented with L-glutamine, pen/strep) or with HT29 CM. HCT116 cells were grown in complete culture medium in 24-well plates until reaching confluence. Subsequently, a scratch was made in a confluent monolayer with a sterile pipet tip, detached cells were removed by washing the well with PBS, 1 ml of fresh medium with the same formulation used for the pretreatment was added to HMEC-1 cells or 1 ml of MDM M0 CM was added to HCT116 cells. A wound healing assay for HMEC-1 cells was performed under standard cell culture conditions in a Spark Multimode Microplate Reader (Tecan, Switzerland) equipped with a humidity cassette. Migration progress was documented by whole-well imaging for 24 h with confluence measurements every 2 h. The wound healing intensity of HCT116 cells was documented using a Nikon Eclipse TE 2000-U microscope immediately and 2, 6, 12 and 24 h after CM addition. Images were analysed using ImageJ 1.53e software and the MRI Wound Healing Tool.

### Microtube formation

HMEC-1 cells were grown in MCDB131 culture medium on 12-well plates to 70–80% confluence. Twenty-four hours prior to the experiment, the medium was changed to MCDB-131 culture medium without EGF and hydrocortisone, and then the cells were preincubated for 3.5 h with 500 nM NMU-9 or 50 ng/ml VEGF-A in MCDB131 medium (supplemented with L-glutamine, pen/strep) or with HT29 CM. Subsequently, the cells were washed with PBS and detached with Accutase (Sigma–Aldrich), and 3 × 10^4^ cells were seeded on 10 mg/ml Matrigel-coated 24-well plates in fresh medium as described above. After 6 h of incubation under standard cell culture conditions, tube formation was documented with a Nikon Eclipse TE 2000-U microscope (Nikon). Images were analysed with ImageJ 1.53e software and the Angiogenesis Analyser Tool, and the mean junction number and total segment length were estimated.

### Statistical analysis

The Shapiro–Wilk test was used to confirm the Gaussian distributions of raw data. Data with a normal distribution are presented as the means and SD; otherwise, medians and interquartile ranges are reported. For unpaired comparisons, Student’s t test or Welch's test for unequal SDs was performed as appropriate to test the differences in normally distributed data between groups. The Mann–Whitney U test was performed to test the differences in data with nonnormal distributions between groups. In the case of multiple comparisons, differences in data departing from a normal distribution were analysed using the Kruskal–Wallis test followed by Dunn’s multiple comparisons test. In the case of relative comparisons to hypothetical values, the Wilcoxon signed-rank test or the one-sample t test was used according to the data distribution.

## Results

### NMU and NMUR1 expression in CRC tissue correlates with the CRC status and patient survival

**Fig. 1:**
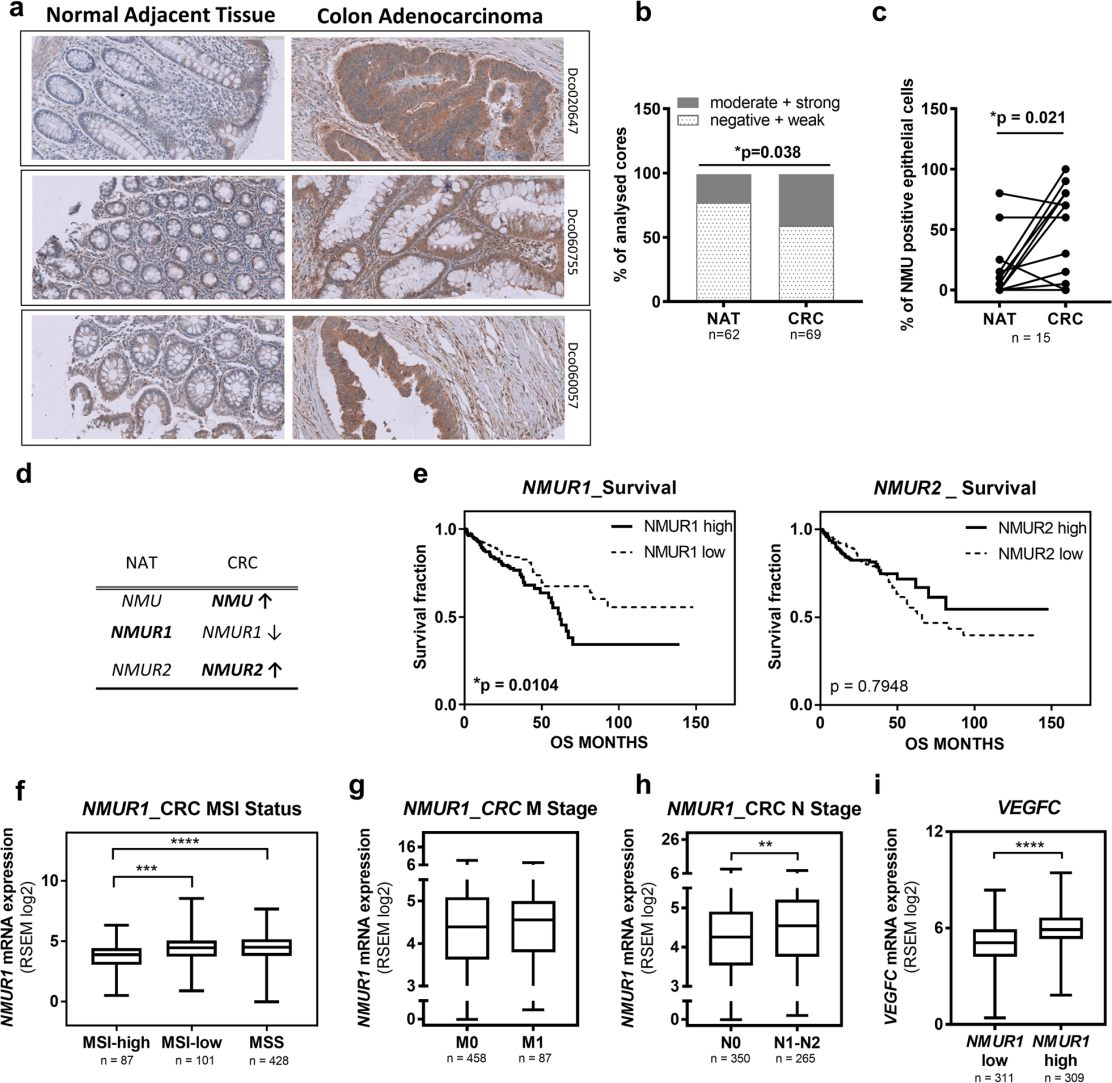
Human CRC tissues—IHC and TCGA data analysis. **a** Tissue samples (grade 1) collected on tissue microarrays stained with anti-NMU antibodies (IHC) and analysed under the microscope (zoom 20x, field 0.3 mm^2^). **b** Percent of all analysed cores with different levels of NMU staining (Fisher’s exact test). **c** Percent of NMU-positive epithelial cells in the analysed CRC tissues, grade 1 (Wilcoxon matched-pairs signed rank test). NAT—normal adjacent tissue. **d** Changes in *NMU* and *NMUR*s expression in CRC tissues compared to normal samples based on the expression levels extracted from TCGA RNA sequencing dataset (published here [14]). **e** Ordered patient data with survival data were divided into *NMUR* low (n = 260) and *NMUR* high (n = 260) expression groups according to the median values, and survival rates were compared between the groups by performing a Kaplan–Meier analysis (log-rank (Mantel–Cox) test, χ^2^ = 6.559, df = 1, *p* = 0.0104). *NMUR1* expression was extracted from TCGA RNA sequencing dataset and compared among CRC tumours with different **f** MSI statuses (Kruskal–Wallis test, *p* < 0.0001, Dunn’s multiple comparisons test, ***p = 0.0008; ****p < 0.0001), **g** M stages (Mann–Whitney test, two-tailed, *p* = 0.4419), **h** N stages (Mann–Whitney test, two-tailed, *p* = 0.0079), and **i** *VEFGC* expression between groups with *NMUR1* low and *NMUR1* high levels according to the median values (Mann–Whitney test, two-tailed, ****p < 0.0001). The boxes represent the interquartile ranges, the horizontal lines in the boxes represent the medians, and the whiskers represent the minimum and maximum values

Our previous analysis of TCGA patient datasets showed higher expression of *NMU* in CRC tissue than in normal adjacent tissue [14]. Here, we validated these results and we detected an increase in NMU peptide levels in cancer tissue samples by performing immunohistochemical staining (IHC) of commercially available tissue microarrays (Fig. 1a, Additional file 3). We analysed cores from 56 normal adjacent tissues (NAT) and from 58 cases of adenocarcinoma. Differences in staining between cores from the same patient were observed, and thus all core results were analysed. Forty-seven adenocarcinoma tissues were matched with cancer-adjacent normal tissue (Additional file 3). We observed moderate/strong NMU staining significantly more often in the tumour cores than in normal adjacent colon tissue (Fig. 1a, b). In paired samples, a significant difference in NMU staining was detected in low-grade tumours (g1) (Fig. 1c).

Unfortunately, the lack of reliable antibodies discriminating between NMUR1 and NMUR2 made identifying NMUR1-positive cells difficult by immunohistochemistry. Hence, we analysed expression data of NMU receptors collected in TCGA. Regarding the *NMUR1* mRNA expression level in CRC tissues, although expression was lower in CRC samples than in normal adjacent tissue (Fig. 1d), patients with high *NMUR1* expression experienced significantly shorter OS (Fig. 1e). We did not observe a correlation between *NMUR2* levels and patient survival (Fig. 1e). Next, we analysed *NMUR1* expression in patients with CRC (Additional file 4) after stratification according to microsatellite stability (MSS—microsatellite stable, MSI—microsatellite unstable) (Fig. 1f), metastasis status classified by the M stage (Fig. 1g) and lymph node invasion status reflected by the N stage (Fig. 1h). The obtained results showed that *NMUR1* expression was frequently elevated in MSI-low and MSS cancers compared with MSI-high cancers. Although *NMUR1* expression was comparable in patients with or without metastasis, its level was significantly increased in cancers with lymph node invasion and correlated with increased expression of *VEGFC* (vascular endothelial growth factor C), an inducer of lymphangiogenesis (Fig. 1i). In summary, high *NMUR1* expression is a feature of CRC with a poor prognosis. To elucidate the mechanisms underlying the poor outcome of CRC with high *NMUR1* expression, we decided to identify the types of NMUR1-positive cells present in the tumour tissue, as the tumour composition is not restricted to cancer cells. Hence, we further searched for cellular markers present in the TME that correlate with *NMUR1* expression.

### High *NMUR1* expression in CRC tissue correlates with the expression of endothelial cell, macrophage and platelet markers

**Fig. 2:**
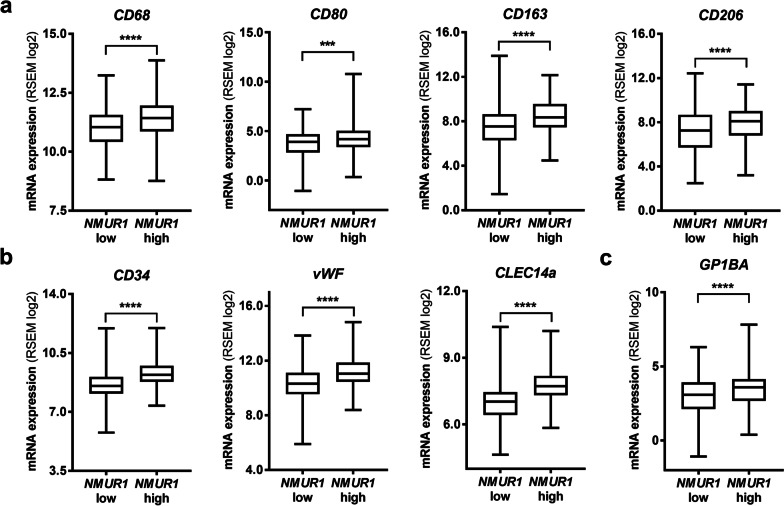
Expression of tumour microenvironment cell markers in human CRC tissues—TCGA data analysis. Ordered patient data were divided into *NMUR1* low (n = 311) and *NMUR1* high (n = 310) expression groups according to the median values. RNA expression levels of cell surface markers of **a** macrophages—*CD68*, M1 macrophage subtype—*CD80* and M2 macrophage subtype—*CD163* and *CD206*; **b** endothelial cells—*CD34*, *vWF*, *CLEC14a;* and **c** platelets—*GP1BA* were analysed in the groups. The boxes represent the interquartile ranges, the horizontal lines in the boxes represent the medians, and the whiskers represent the minimum and maximum values (**a** *CD68* and *CD163* and **b**, **c** unpaired t test with Welch’s correction, two-tailed, ****p < 0.0001; **a** *CD80* and *CD206* Mann–Whitney test, two-tailed, ***p < 0.001, ****p < 0.0001)

*NMUR1* is expressed by CRC cells, as we reported recently [14] and NMUR1 activity supports cancer cell motility, as receptor silencing decreased the migration of HCT116 cells (Additional file 2: Fig. S2). However, according to published data, NMUR1 is also widely expressed by other cell types present in the tumour niche [12]. Thus, using TCGA database, we analysed the expression of molecular markers of macrophages, endothelial cells and platelets in cancer tissues to identify whether the expression of *NMUR1* correlates with these markers. According to the median expression level, the ordered data were divided into low *NMUR1* expression and high *NMUR1* expression groups (Additional file 4). The expression levels of surface markers of macrophages, endothelial cells and platelets were compared between the groups. We detected significantly higher expression of macrophage markers of different phenotypes (Fig. 2a), endothelial cell markers (Fig. 2b) and platelet markers (Fig. 2c) in tissues characterized by high *NMUR1* expression.

Next, we verified whether these cells could be activated by NMU.

### Macrophages and endothelial cells respond to NMU through NMUR1

**Fig. 3:**
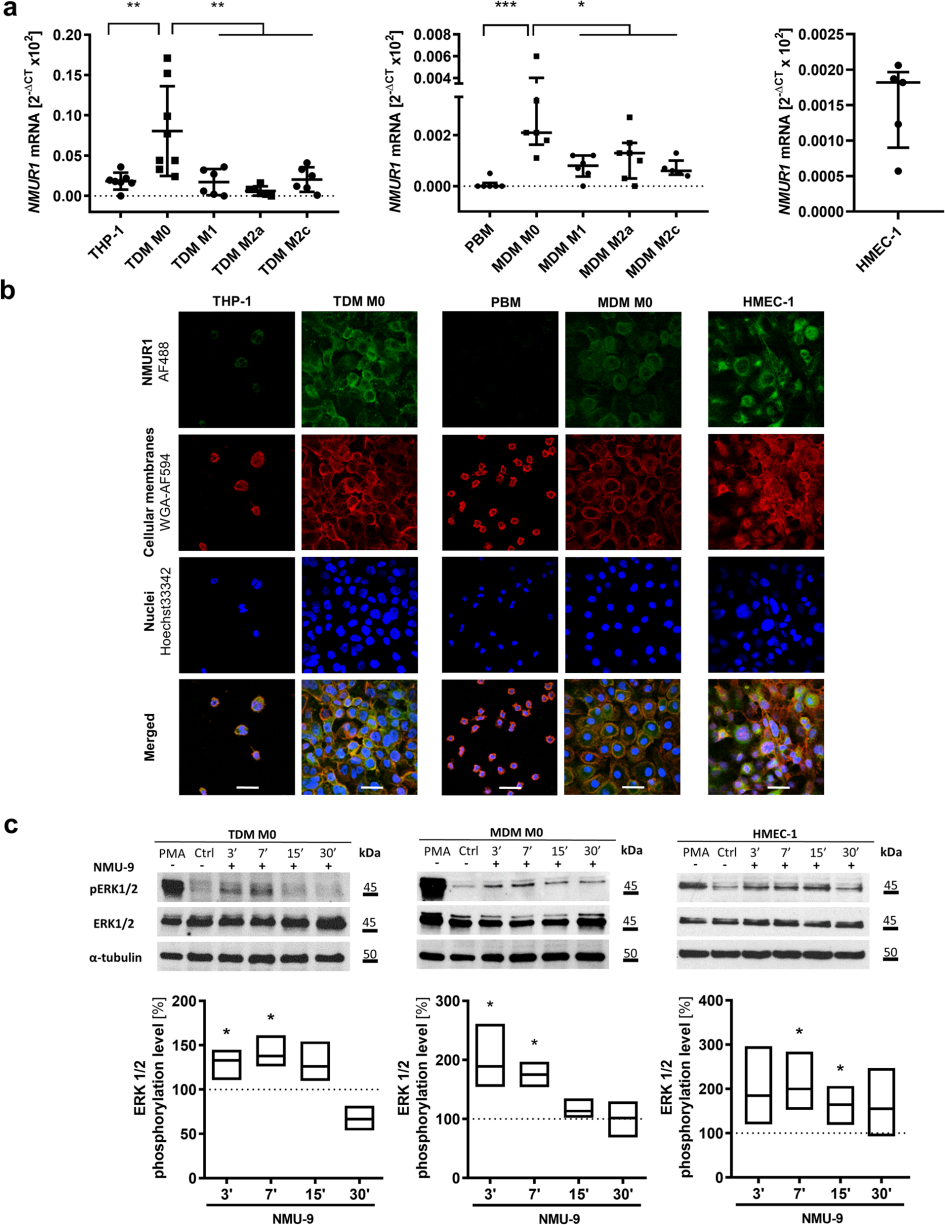
NMUR1 expression and activity in macrophages and endothelial cells. **a** *NMUR1* expression in THP-1 cells and various phenotypes of TDMs (left graph), human PBMs and various phenotypes of MDMs (middle graph) and HMEC-1 cells (right graph). The results are presented as the means with SD (ordinary one-way ANOVA test, Dunnett's multiple comparisons test, *p ≤ 0.05; **p ≤ 0.01, n ≥ 5). **b** NMUR1 protein visualized using confocal microscopy (scale bar = 20 µm). **c** ERK1/2 activation after NMU-9 (250 nM) treatment of TDM M0 (left graph), MDM M0 (middle graph) and HMEC-1 (right graph) cells as analysed using immunoblotting. The image shows a representative result. The bands were quantified using densitometry. The intensity of the pERK1/2 bands was normalized to the respective ERK1/2 and α-tubulin bands. The results are presented as the means with min-to-max ranges (one sample t test, *p ≤ 0.05; n ≥ 3)

We analysed NMU receptors expression in unpolarized TDMs and MDMs, as well as in TDMs and MDMs polarized into the M1, M2a and M2c phenotypes (Additional file 2: Fig. S1). We did not detect *NMUR2* expression. We observed *NMUR1* expression in all phenotypes of macrophages except for PBMs (Fig. 3a), and we showed that unpolarized TDM M0 and MDM M0 cells expressed the highest *NMUR1* levels. Immunofluorescence staining confirmed the presence of NMUR1 on unpolarized macrophages (Fig. 3b). NMUR1 expression was also detected in the human endothelial cell line HMEC-1 (Fig. 3a, b) with no expression of NMUR2. These results were confirmed by immunoblotting (Additional file 2: Fig. S3a). Additionally, we analysed NMU expression in macrophages and endothelial cells, but we did not detect NMU expression (Additional file 2: Fig. S3b, c). Thus, we expect that none of the analysed cells would secrete a significant amount of NMU into the tumour microenvironment; however, they are potentially able to respond to NMU via NMUR1. It is acknowledged that phosphorylation of ERK1/2 kinase (pERK1/2) can be used as a common endpoint measurement for the activation of many classes of G protein-coupled receptors. Thus, we first analysed ERK1/2 activation in cells after NMU-9 treatment and detected significant phosphorylation of ERK1/2 in M0 macrophages and HMEC-1 cells (Fig. 3c). Additionally *NMUR1* silencing decreased ERK1/2 activation in macrophages (Additional file 2: Fig. S4). Next, we used CM from previously characterized [14] CRC cells overexpressing NMU as a source of CRC-released NMU. We analysed the effects of CM from control (HT29-pcDNA) and NMU-secreting (HT29-pcNMU) CRC cells on ERK1/2 activation (Additional file 2: Fig. S5). Components of CM from both control and NMU-secreting clones had the potential to induce ERK1/2 phosphorylation (Additional file 2: Fig. S5a) with increased activation of macrophages by CM from HT29-pcNMU clones (Additional file 2: Fig. S5b). Using Proteome Profiler, we screened the cytokines secreted by control (HT29-pcDNA) and NMU-secreting (HT29-pcNMU) clones and all media contained cancer cells activating cytokines (Additional file 2: Fig. S6, Additional file 5). NMU overexpression additionally induced or increased the secretion of important tumour supporting cytokines which would enhance the observed signal activation.

We confirmed also NMUR1 expression in resting and activated platelets, and interestingly, in platelet microparticles (PMPs) actively engaged in the progression of CRC and other cancer types [18] (Additional file 2: Fig. S7). NMUR1 expression and NMU action in platelets were reported previously [19] and were not further studied here.

As we showed the activation of signalling after NMU treatment of M0 macrophages and endothelial cells, next, we searched for functional changes in analysed cells in response to NMU.

### NMU functions as a chemotactic agent and induces the motility and polarization of macrophages

**Fig. 4:**
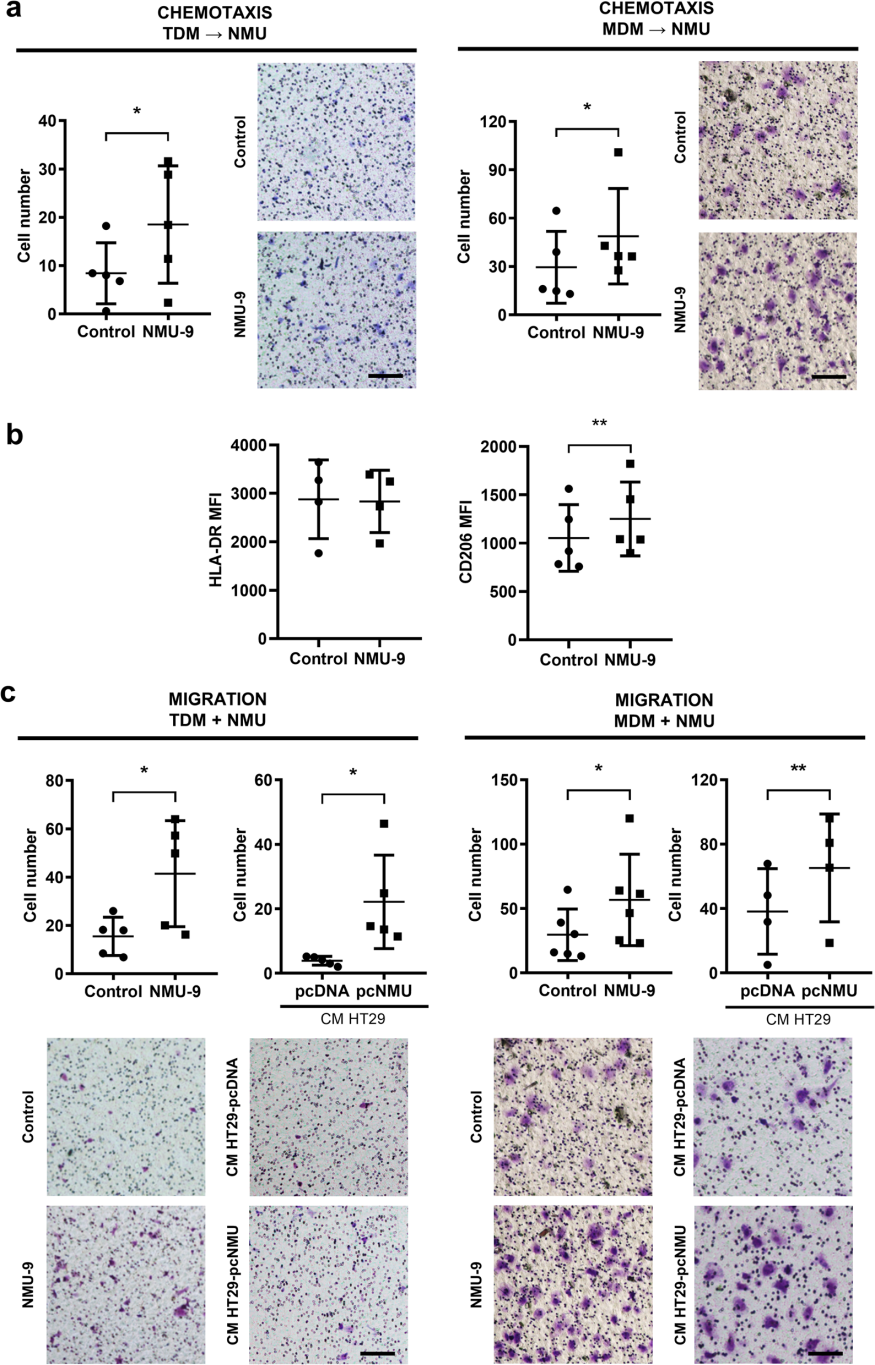
NMU induces the motility of macrophages. **a** TDM and MDM chemotaxis towards NMU-9. **b** Expression of the polarization marker HLA-DR for the M1 phenotype and CD206 for the M2 phenotype on macrophages treated with NMU-9. The results are shown as the means with SD (HLA-DR: paired t test: *p* = 0.6752; n = 4; CD206: paired t test: *p* = 0.0047; n = 5). **c** Migration of TDMs (left panel) and MDMs (right panel) treated with NMU-9 (columns on the left) or CM from HT29 cells secreting NMU (columns on the right). The results are presented as the means with SD (paired t test: *p ≤ 0.05; **p ≤ 0.01; n ≥ 4). The images show representative results (scale bar = 100 µm)

The role of macrophages in CRC progression remains controversial. Cancer cells have been reported to recruit PBMs and tissue-resident macrophages into the tumour niche [20, 21]. We observed that macrophages migrate significantly faster towards medium containing NMU-9 (Fig. 4a), suggesting the chemotactic role of NMU in the tumour environment. As the role of macrophages in the tumour niche depends strongly on their phenotype, we asked whether NMU has the potential to function as a macrophage polarization factor. Interestingly, we detected an increase in the expression of CD206 (a marker of the M2 phenotype) on macrophages treated with NMU-9 (Fig. 4b) but no change in the expression of HLA-DR, a surface marker of M1 macrophages (Fig. 4b). We further examined whether NMU increased the migratory potential of TDMs (Fig. 4c, left panel) and MDMs (Fig. 4c, right panels). We investigated the migration of macrophages after preincubation with NMU-9 (Fig. 4c, left columns in the panel) or with CM from control (HT29-pcDNA) and NMU-secreting (HT29-pcNMU) CRC cells (Fig. 4c, right columns in the panel). We detected increased migration rates of macrophages stimulated with both NMU-9 and CM from HT29-pcNMU clones.

### NMU-9 changes the phenotype of endothelial cells and induces their motility and proangiogenic properties

**Fig. 5:**
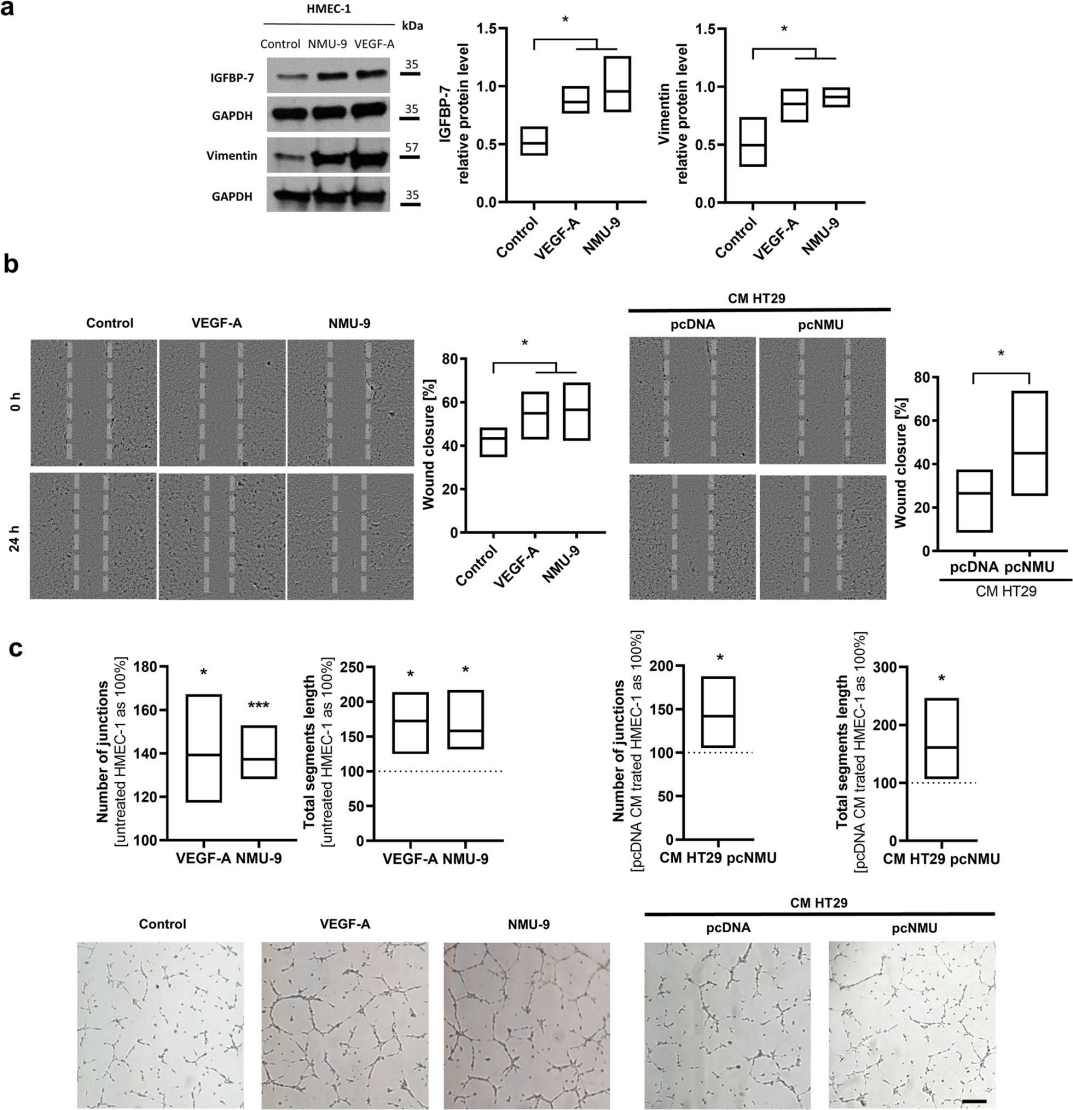
Functional changes in HMEC-1 cells caused by NMU*.* **a** Markers of TECs, IGFBP-7 and vimentin, in HMEC-1 cells treated with NMU-9 (VEGF-A as a positive control) were analysed using immunoblotting. The image shows a representative result. The bands were quantified using densitometry. The intensity of the bands was normalized to the loading control (GAPDH). The results are presented as the means with min-to-max ranges (ordinary one-way ANOVA, Dunnett's multiple comparisons test: *p ≤ 0.05; n ≥ 3). **b** Migration and **c** microtube formation abilities of HMEC-1 cells treated with NMU-9 (left panel) or CM from HT29 cells secreting NMU (right panel) (VEGF-A served as a positive control). **b** The extent of wound closure at 24 h during the experiment was compared with the initial measurement. The results are presented as the means with min-to-max ranges (RM one-way ANOVA, Dunnett’s multiple comparisons test: *p ≤ 0.05; n ≥ 4). The images show representative results. **c** Number of junctions and total length of segments formed by HMEC-1 cells treated with NMU-9 or CM containing NMU were compared with untreated cells or incubated with CM from pcDNA clones. The results are presented as the means with min-to-max ranges (one sample t test: *p ≤ 0.05; ***p ≤ 0.001; n ≥ 4). The images show representative results (scale bar = 100 µm)

Endothelial cells present in the tumour niche gain features of tumour endothelial cells (TECs), and it supports disease progression. NMU-9 treatment of HMEC-1 cells appeared to significantly increase the expression of the TEC markers, insulin-like growth factor-binding protein 7 (IGFBP-7) and vimentin (Fig. 5a). Moreover, functional experiments showed that NMU induced endothelial cell motility (Fig. 5b) and promoted microtube formation in 3D cultures (Fig. 5c).

### NMU-9 alters cytokine secretion by CRC cells, macrophages and endothelial cells

**Fig. 6:**
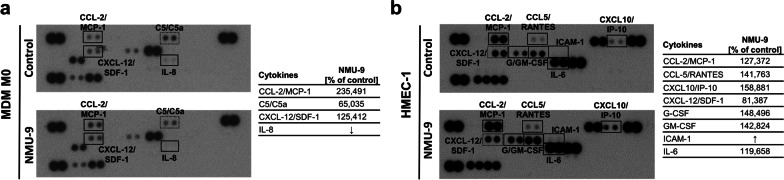
NMU alters cytokine secretion by macrophages and endothelial cells*.* Secretion of cytokines by **a** MDM M0 cells or **b** HMEC-1 cells. The images show representative results. The dots corresponding to individual cytokines were quantified by densitometry and normalized to the reference. The levels of cytokines secreted from **a** NMU-9 (500 nM)-treated MDM M0 cells or **b** NMU-9 (500 nM)-treated HMEC-1 cells were compared with the levels of cytokines secreted from untreated cells (n = 1). Corresponding dots are marked by solid (increase) or broken lines (decrease)

To verify our assumption that NMU modifies the tumour microenvironment by interacting with different cells present in the TME we screened the cytokines secreted by CRC cells overexpressing NMU or by macrophages and endothelial cells after the incubation with NMU-9. We detected changes in cytokine production in all tested cell types (Fig. 6, Additional file 2: Fig. S6, Additional file 5). NMU overexpression in HT29 cells induced or increased the secretion of tumour supporting cytokines (CXCL1/GROa, CXCL10/IP-10 and ICAM-1/CD54, CCL-5/RANTES, IL-8 and IL-1ra) (Additional file 2: Fig. S6). Macrophages treated with NMU-9 secreted elevated levels of CCL-2/MCP-1 and the CXCR4 receptor ligand CXCL12/SDF-1 (Fig. 6a) but decreased amount of IL-8. HMEC-1 cells treated with NMU-9 secreted much higher levels of cytokines that were also elevated in CRC cells and macrophages, namely, CCL-2/MCP1, CCL5/RANTES, CXCL10/IP-10 and ICAM-1/CD54 and G-CSF and GM-CSF (Fig. 6b). For all tested cell types, NMU changed the profile of secreted cytokines.

### NMU-stimulated macrophages increase the motility of HCT116 colon cancer cells.

**Fig. 7:**
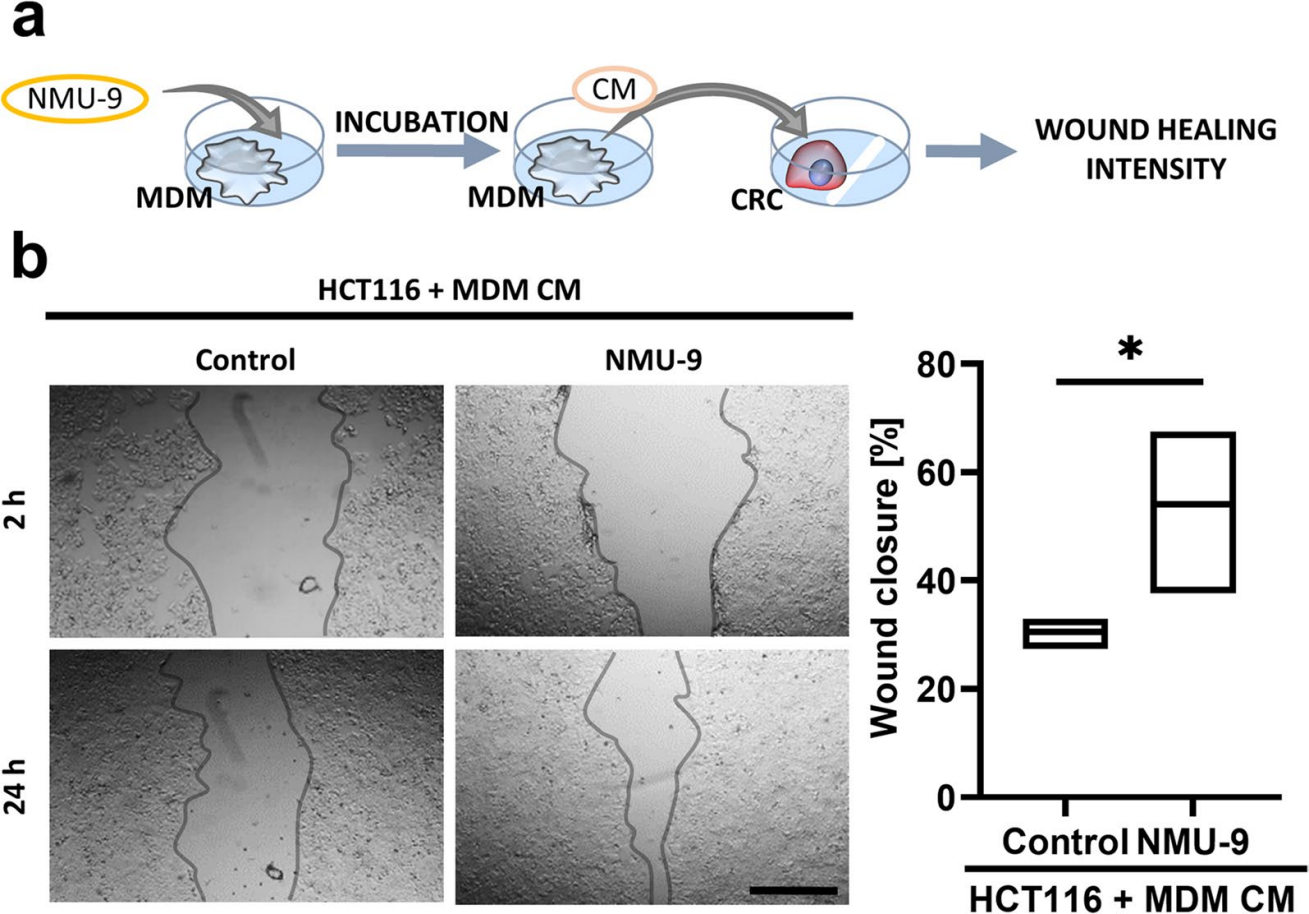
Indirect regulation of CRC cell motility by NMU. Migration of HCT116 cells incubated with CM from untreated MDMs (control) or MDMs treated with NMU-9 (NMU-9). **a** The scheme presents the experimental design. **b** Images show representative results (scale bar = 500 µm). The extent of wound closure at 24 h during the experiment was compared with the initial measurement. The results are presented as the means with min-to-max ranges (paired t test: *p ≤ 0.05; n = 4)

Macrophages abundant in the tumour niche are known for both tumour-promoting and tumour-suppressing actions. We examined whether described above changes in the phenotype of macrophages induced by NMU altered their effect on CRC cell motility. We observed increased migratory properties of HCT116 cells after the incubation with CM from NMU-9-treated macrophages (Fig. 7).

## Discussion

Our previously reported analysis of gene expression data showed significantly elevated *NMU* expression in CRC tissues [14], with *NMU* identified among 10 hub genes closely related to CRC progression [22–24]. These data encouraged us to pursue studies on the importance of NMU activity in CRC. We confirmed the increased level of this peptide in CRC tissue microarrays; however, in paired tissues, a distinct difference in NMU staining between tumour and normal adjacent tissue was observed in low-grade tumours.

NMU binds to the specific receptors NMUR1 or NMUR2, which are unevenly distributed on different types of CRC cells and, as we showed, are related to inducing cancer cell motility [14]. Most of the previously analysed CRC cell lines express increased *NMUR2,* which correlates with perineural invasion in CRC samples [14]. However, our analysis of TCGA data showed that shorter OS of patients and lymph node invasion are related to higher *NMUR1* expression in CRC. This finding was unexpected, as *NMUR1* expression is decreased in CRC tissue compared with NAT, which suggests a tumour-suppressive rather than a tumour-supportive role for the receptor. Unfortunately, data collected in TCGA are the result of the analysis of tumour samples with various levels of “purity” in terms of cancer cells present in the sample (> 50%), and thus the expression data may reflect not only cancer cells but also other TME cellsin the tissue. Interestingly, NMUR1 was also expressed by other cell types potentially present in the TME and known to be active in the tumour niche and on the tumour edges [12]. The analysis of gene expression data showed that CRC tissues with relatively high *NMUR1* expression are also characterized by increased expression of tumour niche cell markers, including macrophages, endothelial cells and platelets. This observation shifted our focus towards NMUR1 activity in cells present in the tumour microenvironment, known as cancer niche modulators [25]. We hypothesized that NMU released by CRC cells influences cancer cells and other NMUR1-positive cells present in the TME, resulting in shorter OS of patients.

First, we confirmed NMUR1 expression in macrophages with different phenotypes, endothelial cells and platelets. We showed receptor-related cellular signalling and then assessed the functional changes caused by NMU, which might lead to cancer progression and worse patient outcomes.

Macrophages and endothelial cells, which are essential components of the TME, have not been previously described as a targets for cancer cell-secreted NMU. Interestingly, NMU appeared to be a chemoattractant for unpolarized macrophages, suggesting that the peptide might recruit macrophages into the TME. A positive correlation between the NMU expression level and macrophage percentage was reported only by Li et al. [26] in hepatocellular carcinoma (HCC) tissue. Macrophages in CRC perform both tumour-suppressive and tumour-promoting functions [27], depending on their phenotype and localization in cancer tissue [28]. However, their prognostic value in CRC is still a matter of debate. A recently published report showed that tumour-associated macrophage (TAM) at the tumour invasive front might play a role in the early stages of CRC progression [28]. Because macrophages actively adapt their phenotype to microenvironmental signals, we asked whether NMU, in addition to increasing recruitment and motility, was potentially involved in inducing the TAM phenotype. After NMU treatment, we detected higher levels of CD206 (M2 macrophage surface marker) and no change in HLA-DR (M1 phenotype marker) expression. We conclude that NMU recruits and induces macrophage polarization towards a mixed phenotype characteristic for macrophages in tumours.

Endothelial cells treated with NMU also change their phenotype, increase the production of IGFBP-7 and vimentin, markers of tumour endothelial cells (TECs), and become more motile with an increased ability to form capillaries. The dysfunctional phenotype of TECs and the “angiogenic switch” allow CRC cells not only to survive and grow but also provide them access to vessels, resulting in metastatic progression and dissemination [29]. NMU seems to be an important regulator of these processes.

Macrophages and endothelial cells, together with cancer cells, were found to form complexes called the tumour microenvironment of metastasis (TMEM) [30] that enable cancer cell intravasation and dissemination in some types of cancers. Because of the proximity of the studied cells in the TME, we analysed the profiles of cytokines secreted by cancer cells, macrophages and endothelial cells stimulated with NMU, which appeared to modify cytokine secretion and favour tumours supporting immunosuppressive conditions [31]. We detected increased secretion of cytokines reported to be promoters of CRC growth in vivo (CCL5 [32] and CCL2 [33]), poor prognostic biomarkers in CRC (CXCL10 [34], CCL2 [35]), promigratory factors (CXCL-12 [36]), and indicators of an immunosuppressive TME (CCL5 [37] and ICAM-1 [38]), along with cytokines with additional proangiogenic (ICAM-1 [38]) and macrophage recruitment potential (CCL2 [39] and CXCL10 [34]). We detected higher amounts of proinflammatory cytokines secreted by NMU-stimulated HMEC-1 cells, including MCP-1, CCL-5, IL-6, G-CSF, GM-CSF and IL-6, and increased ICAM-1 levels, which led us to conclude that NMU elicits a dysfunctional endothelial cell phenotype. As reported by Franses J.W. [40], dysfunctional ECs, a feature of the tumour vasculature, promote proinflammatory signalling, enhancing the invasiveness of cancer cells. As cytokine profiling provides only general information, the particular changes must be verified in further studies.

To determine how NMU might indirectly modulate CRC cells via cytokines produced by macrophages, we treated HCT116 cells with CM from NMU-treated macrophages. HCT116 colorectal cell line is classified as Consensus Molecular Subtype 4 (CMS4) [41] and defined as mesenchymal, it shows intense stromal infiltration and evidence of the epithelial–mesenchymal transition (EMT) with strong autocrine NMU/NMUR1 stimulation [14]. CM from macrophages stimulated with NMU increased the motility of HCT116 cells. The specific cytokines responsible for this effect remain to be verified, but detected CXCL12 was shown by others to be an effective promigratory cytokine [42].

Platelets, which were also tested here, are well described as promoters of CRC metastasis [43] by interacting with various CRCs of different CMS [44]. NMU secreted by CRC cells may act through NMUR1, as shown by Grippi et al. [19], and potentiate platelet activation induced by low ADP or epinephrine concentrations. Thus, NMUR1 present on platelets in the NMU-rich TME might activate them to promote prometastatic conditions. The reason for NMUR1 transport in PMPs remains unknown and will be the subject of our future studies. PMPs have been shown to transfer different proteins [45, 46] or miRNAs [47] into target cancer cells and activate various intracellular signalling cascades, to induce the expression of proteins or to increase the migratory potential of cells, as has been shown for breast cancer [45] and lung cancer [46]. However, the role of NMUR1 transferred by PMPs in cancer progression must be further investigated.

**Fig. 8:**
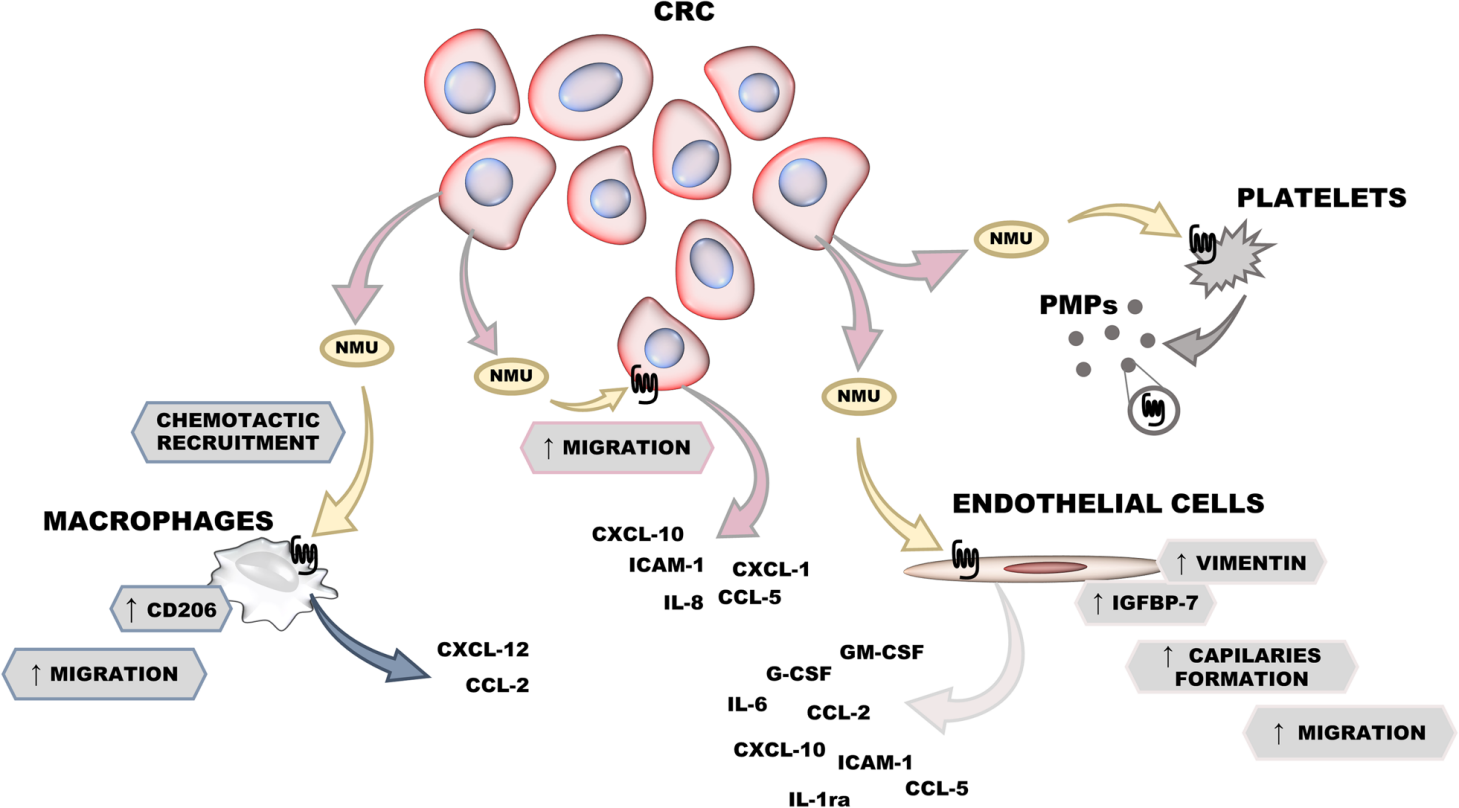
NMU action in the tumour microenvironment in CRC

The presented data showed that NMU induces tumour-promoting phenotypes in cancer cells and NMUR1-expressing macrophages, endothelial cells and platelets present in the TME (Fig. 8). Nevertheless, at the same time, our results generated new, interesting questions, e.g., what is the effect of NMU on other tumour niche cells expressing NMUR1, such as other immune cells or stroma cells, not studied here and what is a potential of NMUR1 as a prognostic marker?

Detection of NMU interaction with cells recruited into the tumour niche shed new light on data obtained from other cancer types where the leading role of NMUR1 was shown. Observation that NMU may play specific roles in modulating the TME, thus facilitating cancer progression was proposed also in neuroblastoma studies [48]. The perspective of NMUR1 as a new prognostic marker or NMU as a new therapeutic target which accessibility in CRC could be modulated conduct additional studies. There are still many unanswered questions about NMU/NMURs expression and signalling in the TME, but current results legitimize further molecular and in vivo studies.

## Conclusions

In summary, our data showed that the shorter survival of patients with CRC characterized by high *NMUR1* expression is mediated by the effect of CRC-secreted NMU and its pro-metastatic activation of NMUR1-positive cancer cells, as well as tumour-infiltrating macrophages, endothelial cells and platelets. NMU potentially modulates TME through the direct and indirect activation of several cellular processes, contributing to cancer progression.

## Supplementary Information

**Additional file 1.** Certificate of cell line authentication.

**Additional file 2.** Supplementary figures and methods.

**Additional file 3.** Tissue microarray (Biomax) description and results.

**Additional file 4.** Gene expression data extracted from TCGA database.

**Additional file 5.** Proteome profiler—raw data.

## Data Availability

The data generated in this study are available within the article and its Additional files. The expression profile data analysed in this study were obtained from TCGA. All microscopy slides and data generated in this study are available upon request.

## Abbreviations

CMS: Consensus molecular subtype
CRC: Colorectal cancer
GPCR: G protein-coupled receptor
NAT: Normal adjacent tissue
NMU: Neuromedin U
NMUR: Neuromedin U receptor
TAM: Tumour associated macrophage
TCGA: The Cancer Genome Atlas
TEC: Tumour endothelial cell
TME: Tumour microenvironment
