# Supplementary material for: Neuromedin U secreted by colorectal cancer cells promotes a tumour-supporting microenvironment
Source: Cell Commun Signal. 2022 Dec 8;20:193. doi: 10.1186/s12964-022-01003-1 (PMC9733105; doi:10.1186/s12964-022-01003-1)
Supplement: Supplementary file 2 — Additional file 1. Certificate of cell line authentication. [file 12964_2022_1003_MOESM2_ESM.pdf]

Eurofins Genomics Europe Applied Genomics GmbH, Anzinger Str. 7 a, D-85560 Ebersberg

Dr. Patrycja Przygodzka
Institute of Medical Biology of PAS, Cellular Signaling
ulica Lodowa 106
93-232, Lodz
Poland

# Certificate
## Cell Line Authentication Test
## Order ID: 11107394206

Report date: 12.05.2021

**Method:**
DNA isolation was carried out from cell pellet (cell layer).
Genetic characteristics were determined by PCR-single-locus-technology.
16 independent PCR-systems D8S1179, D21S11, D7S820, CSF1PO, D3S1358, TH01, D13S317, D16S539, D2S1338, AMEL, D5S818, FGA, D19S433, vWA, TPOX and D18S51 were investigated.
(ASN-0002 core markers are colored grey, Thermo Fisher, AmpFlSTR® Identifiler® Plus PCR Amplification Kit)
In parallel, positive and negative controls were carried out yielding correct results.

**Result:**

| **Client Sample Name** | HT29_IBM | HCT15_IBM | HCT116_IBM | Caco-2_IBM | SW480_IBM | SW620_IBM |
|---|---|---|---|---|---|---|
| **Sample Code** | 21_ZE_000615 | 21_ZE_000616 | 21_ZE_000617 | 21_ZE_000618 | 21_ZE_000619 | 21_ZE_000620 |
| D8S1179 | 10,16 | 15,15 | 11,12,14,15 | 12,14 | 13,13 | 13,13 |
| D21S11 | 29,30 | 29,32.2 | 29,30 | 30,32 | 30,30.2 | 30,30.2 |
| D7S820 | 10,10 | 10,12 | 11,12 | 11,12 | 8,8 | 8,9 |
| CSF1PO | 11,12 | 12,12 | 7,10,11 | 11,11 | 13,14 | 13,14 |
| D3S1358 | 15,17 | 17,17 | 11,12,17,18,19 | 14,17 | 15,15 | 16,16 |
| TH01 | 6,9 | 7,9.3 | 8,9 | 6,6 | 8,8 | 8,8 |
| D13S317 | 11,12 | 8,11 | 10,11,12,13 | 11,13,14 | 12,12 | 12,12 |
| D16S539 | 11,12 | 12,13 | 11,12,13,14 | 12,13 | 13,13 | 9,13 |
| D2S1338 | 19,23 | 17,25 | 16,16 | 17,25 | 17,24 | 17,24 |
| D19S433 | 14,14 | 14,16 | 12,13 | 15,15 | 13,13 | 13,13 |
| vWA | 17,19 | 18,19 | 17,21,22,23 | 16,18 | 16,16 | 16,16 |
| TPOX | 8,9 | 8,11 | 8,9 | 9,11 | 11,11 | 11,11 |
| D18S51 | 13,13 | 11,17 | 16,17,18 | 12,12 | 13,13 | 13,13 |
| AMEL | X,X | X,Y | X,Y | X,X | X,X | X,X |
| D5S818 | 11,12 | 13,13 | 10,11,12 | 12,13 | 13,13 | 13,13 |
| FGA | 20,22 | 22,22 | 17,18,22,23,24 | 19,19 | 24,24 | 24,24 |
| **Database Name** | HT-29 | HCT-15 | HCT 116* | CACO-2 | SW-480 | SW620 |

The table shows the result of the cell line analysis and the comparison with the online database of the DSMZ (http://www.dsmz.de/de/service/services-human-and-animal-cell) and the Cellosaurus database (https://web.expasy.org/cellosaurus). Please note that only the PCR-systems according to ANSI/ATCC standard ASN-0002 were aligned (D5S818, D13S317, D7S820, D16S539, VWA, TH01, TPOX, CSF1PO, AMEL - colored grey).

*The sample could be identified as the stated cell line, although one or several PCR-Systems did show additional signals (see table). These signals could occur due to contamination of the cell line or possible mutations.

**This report was created automatically and is therefore valid without a signature.**

Eurofins Genomics Europe Applied Genomics GmbH, Anzinger Str. 7 a, D-85560 Ebersberg

Dr. Patrycja Przygodzka
Institute of Medical Biology of PAS, Cellular Signaling
ulica Lodowa 106
93-232, Lodz
Country
Poland

eurofins | Genomics

SOP_APG_Zelllinienauthentizität_A04_1.0

Report date: 22.09.2021

**Certificate**
**Cell Line Authentication Test**
**Order ID: 11107589109**

**Method:**
DNA isolation was carried out from cell pellet (cell layer).
Genetic characteristics were determined by PCR-single-locus-technology.
16 independent PCR-systems D8S1179, D21S11, D7S820, CSF1PO, D3S1358, TH01, D13S317, D16S539, D2S1338, AMEL, D5S818, FGA, D19S433, vWA, TPOX and D18S51 were investigated.
(ASN-0002 core markers are colored grey, Thermo Fisher, AmpFlSTR® Identifiler® Plus PCR Amplification Kit)
In parallel, positive and negative controls were carried out yielding correct results.

**Result:**

| **Client Sample Name** | THP-1_IBM | HMEC-1_IBM | HT29_pcDNA_1 | HT29_pcNMU_45 |
|---|---|---|---|---|
| **Sample Code** | 21_ZE_001170 | 21_ZE_001171 | 21_ZE_001172 | 21_ZE_001173 |
| D8S1179 | 10,14 | 11,14 | 8,10,16 | 10,16 |
| D21S11 | 30,31.2 | 29,30 | 29,30 | 29,30 |
| D7S820 | 10,10 | 8,10 | 10,10 | 10,10 |
| CSF1PO | 11,13 | 10,12 | 11,12 | 11,12 |
| D3S1358 | 15,17 | 16,17 | 15,17 | 15,17 |
| TH01 | 8,9.3 | 7,7 | 6,9 | 6,9 |
| D13S317 | 13,13 | 11,12 | 11,11 | 11,12 |
| D16S539 | 11,12 | 11,12 | 11,12 | 11,12 |
| D2S1338 | 17,18 | 19,25 | 19,23 | 19,23 |
| D19S433 | 12.2,13 | 12,17.2 | 14,14 | 14,14 |
| vWA | 16,16 | 15,17 | 17,19 | 17,19 |
| TPOX | 8,11 | 8,11 | 8,9 | 8,9 |
| D18S51 | 13,14 | 17,21 | 13,13 | 13,13 |
| AMEL | X,Y | X,Y | X,X | X,X |
| D5S818 | 11,12 | 12,12 | 11,12 | 11,12 |
| FGA | 24,25 | 21,22 | 20,22 | 20,20 |
| **Database Name** | THP-1 | HMEC-1 | HT29 | HT29 |

The table shows the result of the cell line analysis and the comparison with the online database of the DSMZ (http://www.dsmz.de/de/service/services-human-and-animal-cell) and the Cellosaurus database (https://web.expasy.org/cellosaurus).
Please note that only the PCR-systems according to ANSI/ATCC standard ASN-0002 were aligned (D5S818, D13S317, D7S820, D16S539, VWA, TH01, TPOX, CSF1PO, AMEL - colored grey).

**This report was created automatically and is therefore valid without a signature.**
