# Supplementary material for: Neuromedin U secreted by colorectal cancer cells promotes a tumour-supporting microenvironment
Source: Cell Commun Signal. 2022 Dec 8;20:193. doi: 10.1186/s12964-022-01003-1 (PMC9733105; doi:10.1186/s12964-022-01003-1)
Supplement: Supplementary file 3 — Additional file 2. Supplementary figures and methods. [file 12964_2022_1003_MOESM3_ESM.pdf]

Supplementary Figures

Figure S1

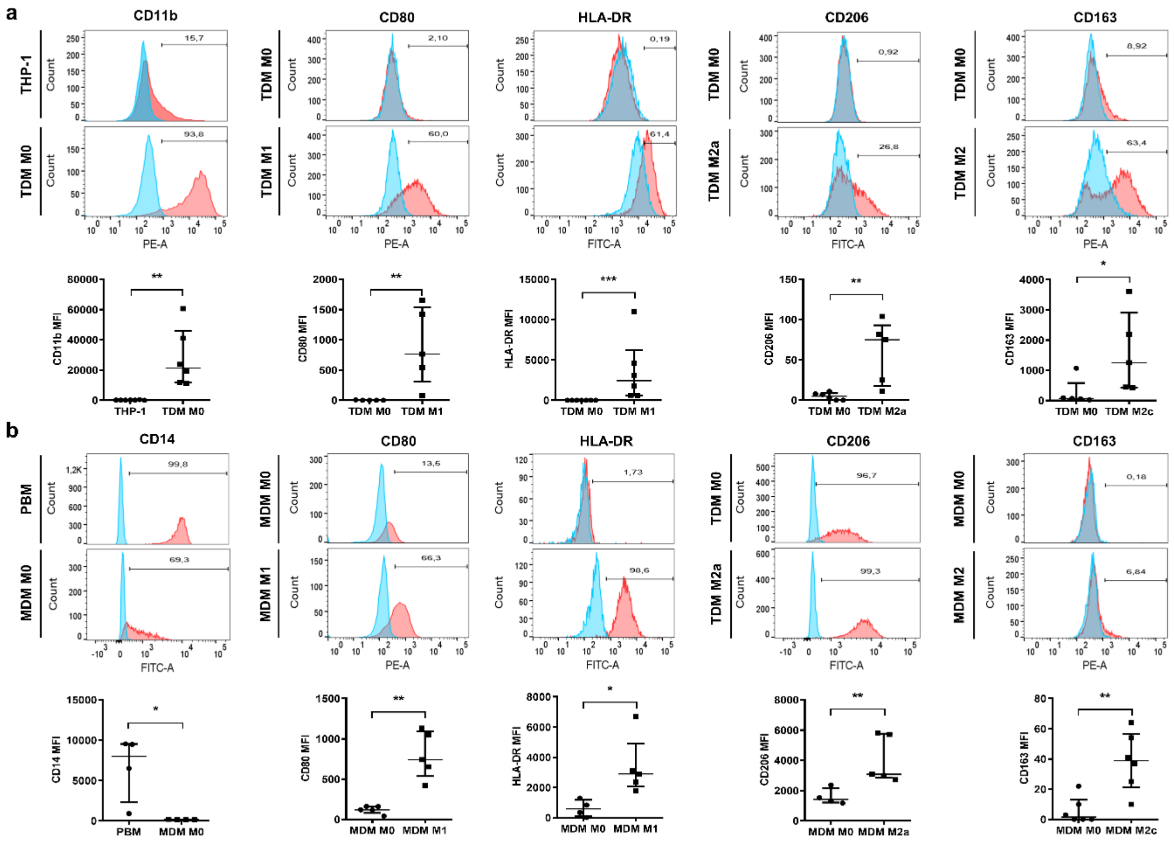

Fig. S1. ***Macrophage cell surface markers expression.*** The level of an appropriate differentiation marker was analysed by flow cytometry in **a** THP-1 cells and THP-1-derived macrophages (TDM M0; CD11b) and **b** human peripheral blood monocytes (PBM) and unpolarised monocyte-derived macrophages (MDM M0; CD14). Polarisation markers, CD80 and HLA-DR for M1 phenotype, CD206 for M2a phenotype and CD163 for M2c phenotype, were analysed in **a** TDM or **b** MDM. The histograms show representative results. The results are shown as the median with interquartile range (Mann-Whitney test: $*p \leq 0.05$; $** \; p \leq 0.01$; $*** \; p \leq 0.001$; $n \geq 4$).

Figure S2

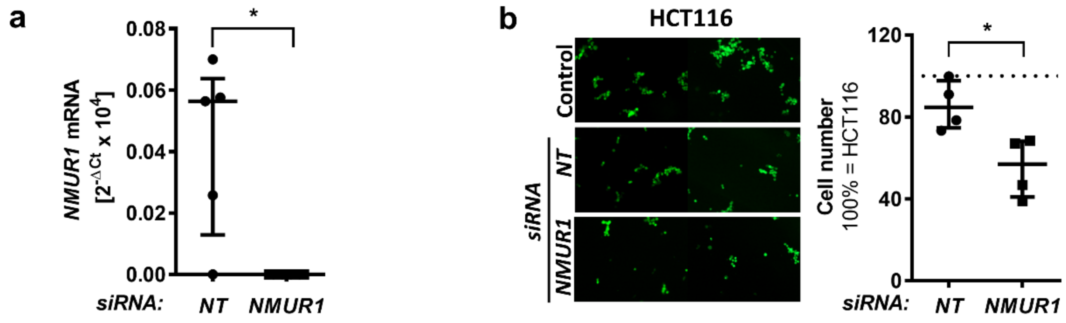

Fig. S2. Autocrine effects of NMU on HCT116 cells with silenced *NMUR1*. a *NMUR1* expression in HCT116 cells transfected with nontargeting (*NT*) or *NMUR1* specific *siRNA* (*NMUR1*). The results presented as the medians with interquartile ranges (one-sample t-test with theoretical mean = 0, *p < 0.05 (two-tailed p = 0.0303), n = 5). **b** The images show representative results from the Transwell migration assay of control and transfected HCT116 cells with incorporated CellTracker™ Green CMFDA dye. The results in the graph are shown as the medians with interquartile ranges (unpaired t test with Welch's correction, *p < 0.05 (two-tailed p = 0.0199), n = 4).

Figure S3

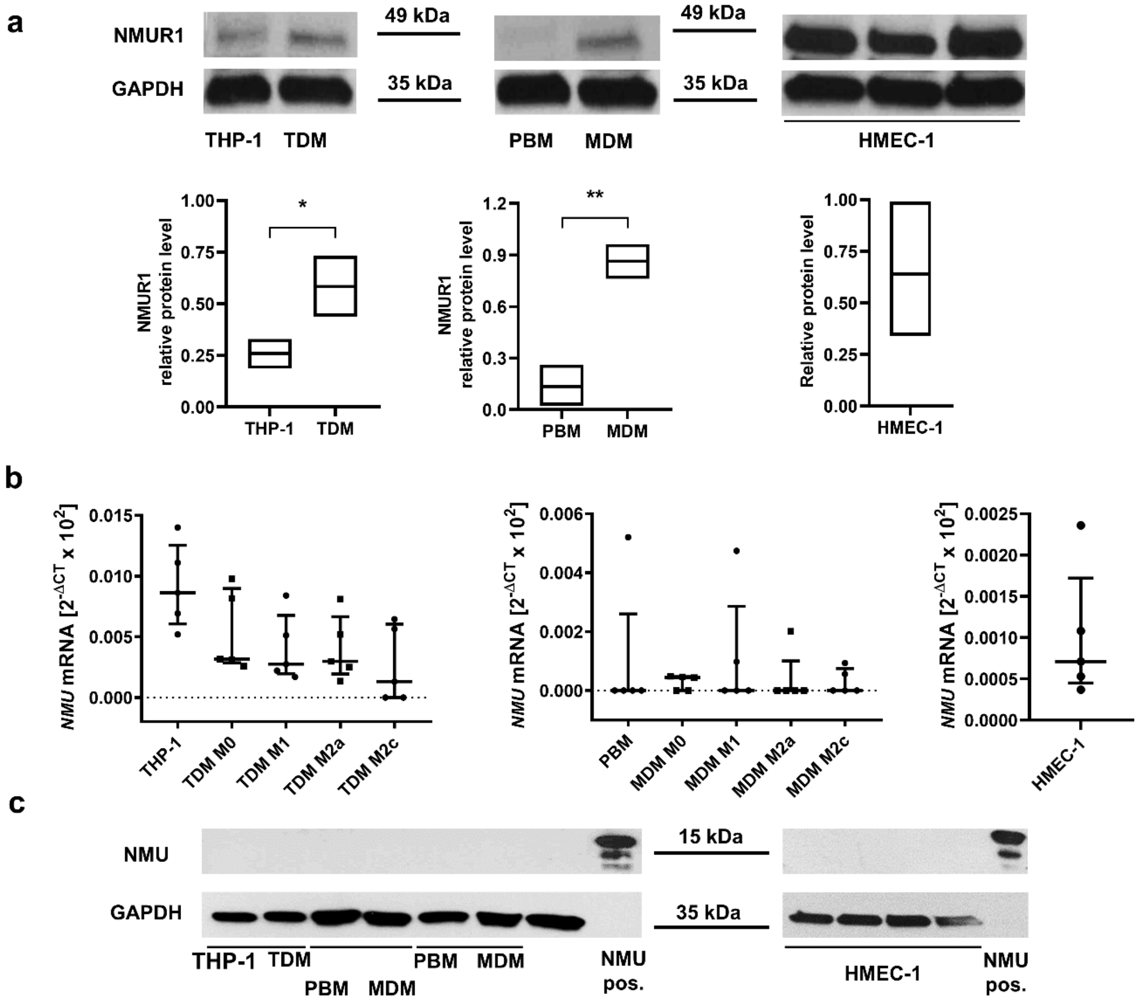

Fig. S3. ***NMUR1 and NMU expression in macrophages and endothelial cells.*** **a** NMUR1 protein level in THP-1 cells and unpolarised THP-1-derived macrophages (TDM) (left graph), human peripheral blood monocytes (PBM) and unpolarised monocyte-derived macrophages (MDM) (middle graph) and HMEC-1 cells (right graph) analysed by immunoblotting. The images show representative results. The bands were quantified by densitometry. The intensity of bands was normalized to loading control (GAPDH). The results are shown as the mean with min-to-max range (One sample t test: *$p \leq 0.05$; ** $p \leq 0.01$; $n \geq 4$). **b** *NMU* mRNA expression in THP-1 cells and various phenotypes of TDM (left graph), PBM and various phenotypes of MDM (middle graph) and HMEC-1 cells (right graph). The results are shown as the median with interquartile range (n = 5). **c** NMU protein identification in THP-1 cells and unpolarised TDM, PBM and unpolarised MDM and HMEC-1 cells analysed by immunoblotting. Commercially available NMU-overexpression lysate was used as NMU positive control (NMU pos.). The images show representative results.

Figure S4

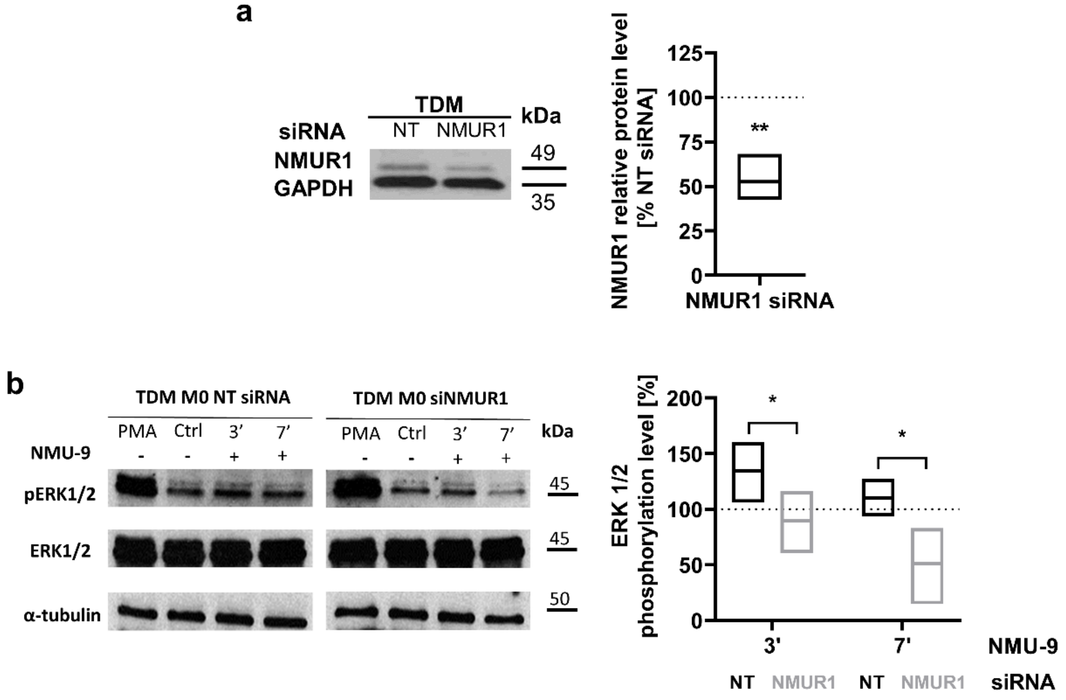

Fig. S4. ***ERK1/2 kinase activation by NMU-9 in macrophages with silenced NMUR1*.** **a** NMUR1 protein level after treatment of TDM M0 with *NMUR1* targeting or non-targeting siRNA analysed by immunoblttting (One sample t test, $**p \leq 0.01$; $n = 4$). **b** Intensity of ERK1/2 kinase activation in TDM M0 with a silenced expression of *NMUR1* after treatment with NMU-9 analysed by immunoblotting. The image shows a representative result. The bands were quantified by densitometry. The intensity of the pERK1/2 bands was normalized to those of the respective ERK1/2 and α-tubulin bands. The results are shown as the mean with min-to-max range (unpaired t test, $*p \leq 0.05$; $n = 4$).

Figure S5

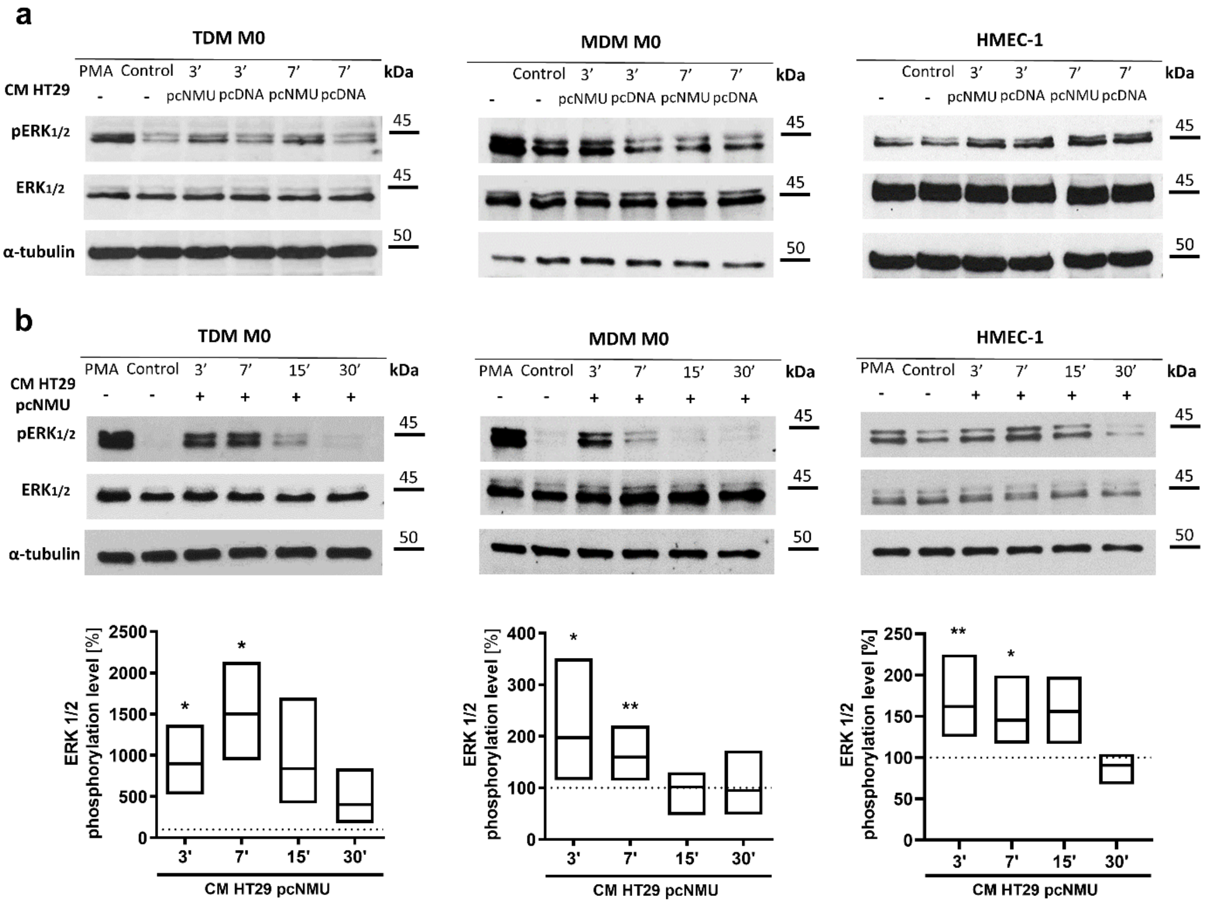

Fig. S5. ***ERK1/2 kinase activation by CRC-secreted NMU in NMUR1 expressing macrophages and endothelial cells.*** **a** Intensity of ERK1/2 kinase activation after treatment of TDM M0 (left graph), MDM M0 (middle graph) and HMEC-1 cells (right graph) with conditioned medium (CM) from HT29 clones secreting NMU (pcNMU) compared to control clones (pcDNA) analysed by immunoblotting. The image shows a representative result. **b** ERK1/2 kinase activation after treatment of TDM M0 (left graph), MDM M0 (middle graph) and HMEC-1 cells (right graph) with NMU secreted by HT29 clones (CM HT29 pcNMU) analysed by immunoblotting. The image shows a representative result. The bands were quantified by densitometry. The intensity of the pERK1/2 bands was normalized to those of the respective ERK1/2 and α-tubulin bands. The results are shown as the mean with min-to-max range (One sample t test, *$p \leq 0.05$; ** $p \leq 0.01$; $n \geq 4$).

Figure S6

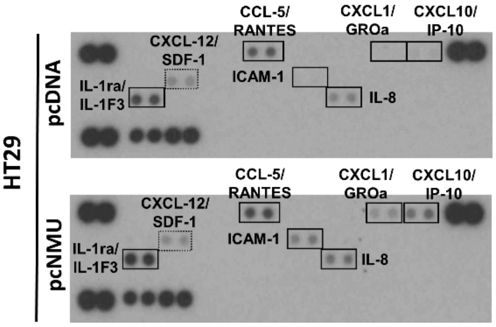

Cytokines	HT29-pcNMU [% pcDNA]
CCL-5/RANTES	128,30
CXCL1/GROa	↑
CXCL10/IP-10	↑
CXCL-12/SDF-1	54,69
ICAM-1	↑
IL-1ra/IL-1F3	116,22
IL-8	120,40

Fig. S6. ***Secretion of cytokines by HT29 clones.*** The image shows a representative result. The dots corresponding to individual cytokines were quantified by densitometry and normalized to the reference. The intensity of cytokine secretion was compared between HT29-pcNMU-overexpressing clones (pcNMU) and control clones (pcDNA), and corresponding dots are marked by solid (increase) or broken lines (decrease).

Figure S7

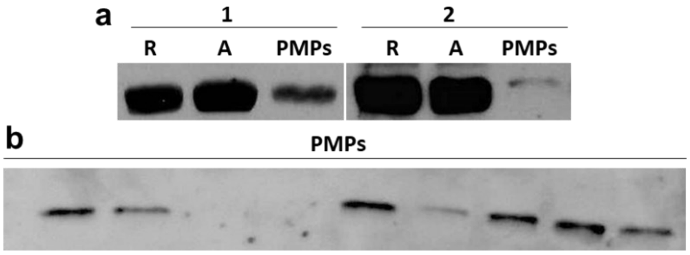

Fig. S7. ***NMUR1 expression in platelets and platelets microparticles (PMPs).*** **a** NMUR1 protein level in (R) resting or (A) thrombin activated platelets and isolated PMPs (two separate isolations) analysed by immunoblotting. **b** NMUR1 protein level in PMPs isolated from activated platelets analysed by immunoblotting (nine separate isolations). The images show representative results. PMPs isolation is described in Supplementary Methods.

Table S1

Surface marker	Isotype	Conjugated fluorophore
CD11b	Mouse IgG1, κ	PE
CD14	Mouse IgG1, κ	FITC
CD80	Mouse C3H/Bi IgG1, κ	PE
HLA-DR	Mouse IgG2a, κ	FITC
CD206	Mouse IgG1, κ	FITC
CD163	Mouse BALB/c IgG1, κ	PE

Supplementary Methods

NMUR1 silencing in HCT116 cells

HCT116 cells were seeded into 24-well plates at a density of 2 x 105 cells/well in medium supplemented with 10% FBS. After 24 h HCT116 cells were transfected with 50 nM NMUR1 siRNA (ON-TARGETplus Human NMUR1 (10316)-siRNA SMARTpool) or nontargeting NT siRNA (ON-TARGETplus Nontargeting Pool) (Dharmacon, Lafayette, CO, USA) using DharmaFECT 2 siRNA Transfection Reagent (Dharmacon) in antibiotic- and serum free- medium according to the manufacturer's instructions. At 24 h posttransfection the medium was replaced with fresh growth medium, and the cells were cultured for 48 h. At 72 h posttransfection mRNA was extracted, and NMUR1 expression was analysed using real-time PCR.

Transwell cell migration assay (HCT116 cells)

The HCT116 cell migration assay was performed using a Transwell insert with an 8.0 µm pore polycarbonate membrane (Thermo Fisher Scientific). HCT116 cells (2 x 105) were incubated with CellTracker™ Green CMFDA Dye (Thermo Fisher Scientific), washed with PBS, resuspended in 200 µl of medium with PS and seeded into Transwells placed in the well with 600 µl of cell culture medium containing pen/strep and 10% FBS. Cells were incubated for 10 h under standard cell culture conditions. Cells remaining inside the chamber were gently removed with cotton swabs, inserts were washed with PBS and the inserts were analysed under the microscope. Images of 5 randomly chosen fields were captured under a Nikon Eclipse TE 2000-U microscope, and migratory fluorescent cells were analysed with ImageJ software.

PMPs isolation

Platelet-derived microparticles (PMPs) were isolated from outdated concentrates of human blood platelets obtained from the Regional Centre of Blood Donation and Blood Treatment in Lodz in accordance with applicable law. Platelets were separated from plasma at 200 x g for 20 min at RT. Then after washing in Tyrode buffer supplemented with EGTA, platelets were resuspended in Tyrode buffer and activated with 2 U/ml thrombin and 2.5 mM CaCl2 for 20 min on rotary shaker. PMPs were isolated after centrifugation of activated platelets for 1500 x g for 20 min at RT followed by ultracentrifugation of resulting supernatant at 100,000 x g for 2h at 4°C. The obtained pellet of PMPs was resuspended in PBS and stored at -80°C until further analysis.
